# Direct or Indirect Action? Mechanisms of the Antiatherosclerotic Effects of Glucagon‐Like Peptide‐1 Receptor Agonists

**DOI:** 10.1155/cdr/8388511

**Published:** 2026-05-13

**Authors:** Michał Zawadzki, Monika Karczewska-Kupczewska

**Affiliations:** ^1^ Department of Internal Medicine and Metabolic Diseases, Medical University of Białystok, Białystok, Poland, umb.edu.pl; ^2^ Department of Metabolic Diseases, Medical University of Białystok, Białystok, Poland, umb.edu.pl

**Keywords:** atherosclerosis, cardiovascular disease, endothelial dysfunction, GLP-1 receptor agonists, inflammation

## Abstract

Cardiovascular diseases (CVD) remain the leading cause of global mortality. Glucagon‐like peptide‐1 receptor agonists (GLP‐1 RAs) significantly reduce major adverse cardiovascular events in patients with and without Type 2 diabetes, offering benefits that extend beyond glycemic control. This review summarizes proposed multifactorial mechanisms underlying these effects, categorizing them into indirect and direct pathways. Indirect mechanisms include, among others, improvements in lipid metabolism, insulin sensitivity, blood pressure, and renal protection. In addition to the previously described effects, GLP‐1 RAs may exert direct vascular actions, including anti‐inflammatory actions, suppression of oxidative stress, improved endothelial function, modulation of macrophage polarization, reduction of adhesion molecule expression, and inhibition of vascular smooth muscle cell proliferation and migration, as well as downregulation of matrix metalloproteinases and plasminogen activator inhibitor‐1. Collectively, these processes contribute to plaque stabilization and reduced atherothrombotic risk. Although clinical evidence for cardiovascular risk reduction of GLP‐1 RAs is robust, mechanistic insights are predominantly derived from preclinical or animal models. Therefore, further functional studies in humans are warranted to clarify the direct antiatherogenic actions of GLP‐1 RAs.

## 1. Introduction

Despite the globally sustained decline in the incidence and mortality of cardiovascular diseases (CVD) over recent years, CVD remain the leading cause of death worldwide, primarily due to the development of serious complications such as ischemic stroke, ischemic heart disease (IHD), and heart failure (HF) [1]. The continuous aging of populations in developed countries, along with the increasing prevalence of lifestyle‐related conditions such as obesity and Type 2 diabetes (T2D), has contributed to the persistently high number of patients affected by CVD, currently estimated at over 500 million globally [1]. Although several classes of drugs with proven antiatherosclerotic effects are currently available (e.g., statins, ezetimibe, fibrates, and pro‐protein convertase subtilisin/Kexin 9 [PCSK9] inhibitors), these data underscore the need to explore new strategies for the prevention and treatment of CVD.

Human glucagon‐like peptide‐1 receptor agonists (GLP‐1 RAs) began to be extensively studied in the 1980s and 1990s. One of the first substances in this group, exenatide, was isolated from the venom of lizards belonging to the Helodermatidae family [2]. Over the following years, the group of GLP‐1 RAs expanded to include seven compounds, which can be categorized into short‐acting agents (exenatide and lixisenatide) and long‐acting agents (liraglutide, dulaglutide, semaglutide, albiglutide, and efpeglenatide). However, it should be noted that albiglutide and efpeglenatide are currently not commercially available. The antidiabetic effects of GLP‐1 RAs were quickly recognized, primarily due to their ability to lower blood glucose levels by stimulating glucose‐dependent insulin secretion from pancreatic beta cells [3]. This mechanism helps prevent drug‐induced hypoglycemia, a common adverse effect of other antidiabetic medications such as sulfonylureas. The glucose‐lowering effect of GLP‐1 RAs is also mediated by the inhibition of excessive glucagon secretion from pancreatic alpha cells, thereby reducing hepatic gluconeogenesis and glycogenolysis [3]. In addition, activation of glucagon‐like peptide‐1 receptors (GLP‐1Rs) located in the gastrointestinal (GI) tract results in delayed gastric emptying and relaxation of intestinal smooth muscle, further reducing postprandial glycemia and suppressing appetite, ultimately leading to weight loss [3, 4]. The effects of GLP‐1 RAs on the GI tract are also mediated by the central nervous system, particularly through modulation of vagal nerve activity, which regulates intestinal motility [5–8]. Moreover, GLP‐1 RAs act via GLP‐1Rs located in the hypothalamus, brainstem, and hippocampus and mesolimbic system, thereby exerting anorexigenic effects [6–8]. Furthermore, a number of studies have demonstrated neuroprotective, procognitive, and anti‐inflammatory effects in the brain, making GLP‐1 RAs promising candidates for the treatment of neurodegenerative diseases [9]. However, in recent years, scientific attention has increasingly focused on the combined effects of GLP‐1 RAs on the cardio‐renal‐metabolic (CRM) syndrome, representing the most clinically significant benefits of this drug class.

Currently, GLP‐1 RAs are recommended in the treatment of T2D and obesity by major diabetes associations [10, 11]. These agents hold a particularly important role in the management of T2D in patients at high CV risk. Their beneficial effects in reducing CV risk in T2D have been demonstrated in large cardiovascular outcome trials (CVOTs). In patients with T2D, studies such as SUSTAIN‐6 (semaglutide), LEADER (liraglutide), and REWIND (dulaglutide) provided evidence of a reduction in major adverse cardiovascular events (MACE) of up to 26% in the groups treated with GLP‐1 RAs [12–14]. Similar results have been observed for initially employed GLP‐1 RAs: albiglutide and efpeglenatide, both of which significantly reduced CV risk in patients with T2D, despite only moderate weight loss being achieved [15, 16]. Additional insights were provided by randomized clinical trials conducted in patients with obesity, such as the SELECT trial (semaglutide), which demonstrated reductions in CV risk factors and a 20% decrease in MACE compared with placebo in patients treated with GLP‐1 RAs [17]. These studies, conducted in patients without T2D, excluded the confounding influence of hyperglycemia on CV risk factor development. Notably, in the SELECT trial, a reduction in 3‐point MACE was observed early on, even before significant weight loss occurred. These findings could suggest that the antiatherosclerotic effects of GLP‐1 RAs extend beyond their glucose‐lowering and weight‐reducing properties. Further support for these observations comes from the recently published real‐world SCORE study, conducted in a population without diabetes, which used semaglutide at a dose of 2.4 mg and reported an unprecedented 57% reduction in 3‐point MACE [18].

Additional evidence for the antiatherosclerotic potential of GLP‐1 RAs is provided by experimental studies in animal models. As early as 2011, Nagashima et al. [19] demonstrated that liraglutide inhibits atherosclerotic lesion development and macrophage infiltration in the aortic wall of apolipoprotein E‐deficient (ApoE−/−) mice, findings that were subsequently confirmed in other studies. The mechanisms underlying the antiatherosclerotic effects of GLP‐1 RAs remain unclear and have been insufficiently investigated in human populations. These effects may be related not only to weight loss and the normalization of metabolic disturbances (e.g., hyperglycemia) but also to direct actions. Therefore, the aim of this paper is to discuss the potential points of atherogenesis inhibition by these agents, based on the most recent scientific literature.

## 2. Suggested Indirect Antiatherogenic Effect of GLP‐1R Agonists

### 2.1. Influence on Carbohydrate and Lipid Metabolism

The beneficial effects of GLP‐1 RAs on CV risk have been suggested to be mediated, at least in part, by the reduction of hyperglycemia in patients with T2D [20]. Hyperglycemia promotes the formation of advanced glycation end‐products (AGEs), which accumulate in tissues and contribute to endothelial activation and inflammatory responses [21]. The interaction of AGEs with their receptor (RAGE) activates intracellular signaling pathways, including nicotinamide adenine dinucleotide phosphate (NADPH) oxidase, leading to increased generation of reactive oxygen species (ROS) and the development of oxidative stress [22]. Oxidative stress, defined as an imbalance between the production of ROS and antioxidant defense mechanisms, plays a key role in endothelial dysfunction [23]. However, many glucose‐lowering agents have demonstrated significant effects on glycemic control without producing a comparable reduction in CV risk to that observed with GLP‐1 RAs.

Furthermore, obesity and T2D are often accompanied by atherogenic dyslipidemia, characterized by an excess of modified low‐density lipoprotein (LDL) particles, such as small dense LDL (sdLDL), very low‐density lipoproteins (VLDL), triglycerides (TG), and decreased levels of high‐density lipoproteins (HDL). These abnormalities contribute to intracellular lipid accumulation and the development of atherosclerotic plaques [24].

Numerous clinical studies have demonstrated the lipid‐lowering effects of GLP‐1 RAs [25–28]. Short‐term treatment with GLP‐1 RAs has been shown to improve postprandial lipid profiles, particularly by reducing TG and VLDL levels both in patients with T2D and in individuals with obesity but without diabetes [29]. Importantly, similar improvements in fasting lipid parameters, additionally with a decrease in LDL concentrations, have been observed after several months of therapy [30]. Although these effects are partly attributed to reduced caloric intake, some clinical studies involving T2D patients treated with liraglutide have suggested additional mechanisms, such as a reduction in apolipoprotein B48 levels and enhanced lipid catabolism mediated by apolipoprotein B100‐containing lipoproteins [31, 32].

Treatment with liraglutide in patients with T2D has also been shown to reduce hepatic and adipose tissue expression of the PCSK9 gene both in vivo and in vitro [31]. At the same time, an upregulation of hepatic low‐density lipoprotein receptor (LDL‐R) expression is observed [31]. Since PCSK9 is a key regulator of LDL‐R degradation, its downregulation may indirectly enhance LDL‐R availability on hepatocyte surfaces, thereby promoting increased clearance of circulating LDL.

### 2.2. Effects on Insulin Sensitivity and Adipose Tissue

Insulin resistance (IR), defined as an impaired biological response to insulin that affects the regulation of carbohydrate, lipid, and protein metabolism, as well as endothelial function, is a key factor in the pathophysiology of T2D. Numerous studies have demonstrated that insulin possesses anti‐inflammatory, antiapoptotic, and vasodilatory properties [33, 34]. IR contributes to the development of atherosclerosis by promoting chronic low‐grade inflammation, hyperinsulinemia, atherogenic dyslipidemia, and decreased nitric oxide (NO) synthesis [35]. One of the effects of GLP‐1 RAs is the improvement of insulin sensitivity, which has been demonstrated in clinical trials using the hyperinsulinemic–euglycemic clamp technique—the gold standard for assessing insulin sensitivity [36]. This effect appears to be mediated primarily through weight loss; however, emerging evidence suggests the existence of direct pathways that may independently contribute to these mechanisms [37].

Dysfunctional adipose tissue plays a significant role in the development of IR [38]. These dysfunctions include impaired lipid storage, disrupted adipokine secretion, and enhanced inflammatory activity. GLP‐1 RAs influence cardiovascular risk in part through their effects on adipose tissue. Agents in this class reduce the volume of total body fat, including visceral, subcutaneous, epicardial, and hepatic fat. However, the most clinically relevant reductions are observed in visceral and epicardial fat depots, which are strongly associated with cardiometabolic risk [39–41]. The reduction of these fat depots is associated with improvements in body weight, glycemic control, and lipid profile, ultimately leading to a decrease in CV risk.

GLP‐1 RAs have been shown to induce a phenotypic shift in adipose tissue toward a less inflammatory state. This effect is mediated, at least in part, through the suppression of nuclear factor kappa B (NF‐*κ*B) signaling and modulation of the Jun N‐terminal kinase (JNK) pathway in adipocytes, leading to decreased production of proinflammatory cytokines such as interleukin‐6 (IL‐6) and tumor necrosis factor‐*α* (TNF‐*α*) [42, 43]. By mitigating these inflammatory processes, GLP‐1 RAs may attenuate the chronic low‐grade inflammation that represents a hallmark of CVD. These indirect adipose tissue–related effects are regarded as a key mechanism underlying the observed reduction in MACE in large CVOTs involving GLP‐1 RAs [44–46].

### 2.3. Effect on Hypertension

Another important component of metabolic syndrome is arterial hypertension, which contributes to mechanical damage of the vascular endothelium and increased lipid accumulation within atherosclerotic plaques. Meta‐analyses involving both patients with and without T2D have demonstrated the antihypertensive effects of GLP‐1 RAs, particularly in reducing systolic blood pressure [47, 48]. This effect has been observed in patients with baseline hypertension as well as in normotensive individuals. However, the exact mechanism underlying the antihypertensive action of GLP‐1 RAs remains unclear.

Although the blood pressure–lowering effect of weight loss is well established, there is insufficient evidence to confirm a direct hypotensive effect of GLP‐1 RAs. In vitro experimental studies have shown no relaxation of preconstricted mouse aortic rings following liraglutide exposure [49]. However, the same study demonstrated an indirect vasodilatory effect of liraglutide mediated by atrial natriuretic peptide (ANP) secretion, which is dependent on GLP‐1R activation in cardiac muscle.

Another study in normotensive rats treated with exenatide revealed the induction of diuresis and natriuresis through inhibition of sodium reabsorption in the proximal renal tubules and an increase in estimated glomerular filtration rate (eGFR) [50]. Elucidating the precise mechanisms of the antihypertensive effects of GLP‐1 RAs remains a challenge due to difficulties in extrapolating animal model findings to the human population and the widespread use of oral antihypertensive agents, which may confound the results of human studies.

### 2.4. Influence on Renal Function

The American Heart Association (AHA) emphasizes that chronic kidney disease (CKD), particularly in the presence of albuminuria or reduced eGFR, is associated with a progressively increased incidence of major cardiovascular events and cardiovascular mortality [51]. This phenomenon is attributed to the proinflammatory and prothrombotic milieu that accompanies CKD [52]. Additionally, uremic toxins promote endothelial dysfunction and the formation of unstable atherosclerotic plaques [52].

GLP‐1 RAs reduce cardiovascular risk also through their beneficial effects on renal function. Large meta‐analyses and randomized controlled trials have shown that GLP‐1 RAs reduce the risk of composite kidney endpoints, including kidney failure, sustained decline in eGFR, or death due to kidney disease by approximately 17%–18%, while also reducing MACE incidence by 13%–14% and all‐cause mortality by 12% in patients with T2D [53–58]. These agents reduce proteinuria and attenuate the decline in eGFR, thereby delaying the development of diabetic kidney disease, an important risk factor for cardiovascular morbidity and mortality [53]. The AHA highlights that GLP‐1 RAs such as liraglutide and semaglutide reduce the risk of onset and progression of CKD, primarily by lowering the incidence of new‐onset macroalbuminuria [59]. These nephroprotective effects contribute to improved cardiovascular outcomes in high‐risk populations.

Although the renoprotective effect of GLP‐1 RAs is less pronounced compared with sodium‐glucose co‐transporter 2 (SGLT2) inhibitors, their favorable impact on cardiovascular outcomes remains clinically relevant across various patient subgroups, including those with established CKD [60]. Proposed mechanisms underlying this renoprotective action include modulation of CKD risk factors such as hypertension, hyperglycemia, and obesity [61]. Additionally, direct nephroprotective effects have been suggested, including attenuation of inflammation, oxidative stress, and renal fibrosis [61].

## 3. Suggested Direct Mechanisms of Antiatherogenic Action of GLP‐1R Agonists

The significant impact of GLP‐1 RAs on the reduction of MACE has recently attracted considerable scientific interest, and ongoing efforts aimed at explaining the underlying mechanisms. Studies demonstrating the expression of GLP‐1R in extrapancreatic tissues, such as the vascular endothelium, vascular smooth muscle, and immune cells, suggest that activation of this receptor may play a crucial role in the direct antiatherogenic effects of GLP‐1 RAs [62, 63]. GLP‐1 RAs, through activation of the GLP‐1R—a class B G protein–coupled receptor (GPCR) that primarily couples to the Gs protein—lead to increased cyclic adenosine monophosphate (cAMP) levels [64]. According to available studies, this mechanism may trigger various intracellular signaling cascades, primarily via the cAMP/protein kinase A (PKA), phosphoinositide 3‐kinase/protein kinase B (PI3K/Akt), AMP‐activated protein kinase (AMPK), mitogen‐activated protein kinase (MAPK) [65, 66]. Evidence, predominantly from experimental studies, suggests that activation of these pathways leads to increased NO production, reduced oxidative stress, and improved endothelial function [65, 66].

Atherogenesis is a multifactorial process in which critical roles are played by interactions among endothelial cells, monocytes, macrophages, vascular smooth muscle cells (VSMCs), and T lymphocytes [67]. The development of atherosclerotic plaques begins with the adhesion of mononuclear leukocytes to the endothelium, driven by increased expression of adhesion molecules—a phenomenon known to be enhanced under conditions of IR. Monocytes then migrate into the intimal layer of arteries in response to chemotactic cytokines, where they differentiate into macrophages. These macrophages, upon engulfing modified lipoproteins, transform into foam cells that produce ROS, matrix metalloproteinases (MMPs), proinflammatory cytokines, and procoagulant factors. Moreover, studies suggest that hyperinsulinemia, via activation of MAPK, promotes the secretion of coagulation factors, which are also synthesized by foam cells [68].

### 3.1. Influence on Inflammation

Already in the second half of the 20th century, a link between CVD and inflammation was recognized [69, 70]. Initial studies in animal models, including monkeys, pigs, and rabbits, demonstrated the antiatherogenic effects of glucocorticoids, immunosuppressive agents, and antihistamines. Patients with chronic inflammatory diseases such as psoriasis and rheumatoid arthritis have been shown to be at increased risk of developing CVD [71, 72]. Specifically, in patients with systemic lupus erythematosus, the elevated CV risk is closely linked to subclinical atherosclerosis, a process potentially mediated by increased IR [73]. A clear association also exists between T2D, obesity, IR, and chronic low‐grade inflammation, which contributes to increased CV risk.

It has been suggested that GLP‐1 RAs may inhibit atherogenesis in part by modulating specific steps of the inflammatory process within the atherosclerotic plaque. As early as 2010, exendin‐4 was shown to suppress monocyte adhesion to the endothelium of the thoracic aorta and attenuate atherosclerotic lesions in ApoE−/− mice [74]. Using positron emission tomography (PET), Jensen et al. demonstrated that semaglutide reduced vascular uptake of tracers targeting activated macrophages and cellular metabolism in an atherosclerotic rabbit model [75]. However, researchers point out that the observed effects may be, at least in part, dependent on the reduction in weight gain induced by GLP‐1 RAs.

Current clinical and experimental studies indicate that GLP‐1RAs may exert a direct effect on oxidative stress. Evidence derived from a meta‐analysis of randomized controlled trials suggests that GLP‐1RAs significantly reduce levels of oxidative stress biomarkers, such as malondialdehyde (MDA), in patients with T2D compared with placebo or standard therapy [76]. The underlying mechanisms may involve both the direct inhibition of ROS production and the activation of antioxidant pathways, for example through the translocation of the nuclear transcription factor erythroid 2p45‐related factor (Nrf2) and increased activity of glutathione‐related enzymes observed in pancreatic *β*‐cells [77, 78]. GLP‐1R activation has also been shown to attenuate oxidative stress via the AMPK/sterol regulatory element‐binding protein 1 (SREBP1) signaling pathway—an important mediator of vascular inflammation in atherosclerosis [79].

In another study involving patients with T2D, a reduction in serum monocyte chemoattractant protein‐1 (MCP‐1) levels was observed after 26 weeks of treatment with either dulaglutide or semaglutide, independent of changes in body mass index (BMI), body weight, or lipid profile [80]. MCP‐1 is a cytokine strongly involved in macrophage recruitment to the atherosclerotic plaque, and elevated MCP‐1 levels are associated with acute coronary syndromes and CVD‐related mortality [81]. However, the study did not rule out confounding effects from hyperglycemia or the concurrent use of anti‐inflammatory drugs such as statins and fibrates, and thus the results should be interpreted with caution.

In a study using murine macrophages, Hou et al. showed that both liraglutide and dulaglutide promoted macrophage polarization from the proinflammatory M1 phenotype to the anti‐inflammatory M2 phenotype [82]. Moreover, both agents reduced the expression of interleukin‐1 beta (IL‐1*β*) and TNF‐*α* in cocultured human umbilical vein endothelial cells (HUVECs), although dulaglutide required a longer exposure time to elicit these effects.

Similar results were observed in a study involving ApoE−/−Irs2+/− mice (a model of atherosclerosis and IR), where lixisenatide enhanced the activation of signal transducer and activator of transcription 3 (STAT3), a key factor in promoting M2 macrophage differentiation [83]. However, it should be noted that in CVOT evaluating lixisenatide did not demonstrate a reduction in cardiovascular risk [84]. In a Danish study using transgenic LDLR−/− mice treated with liraglutide for 4 weeks, expression of genes involved in leukocyte recruitment to the vascular wall—including C‐C chemokine receptor Type 5 (CCR5), fractalkine, and MCP‐1—was reduced [85]. However, this study did not observe a reduction in atherosclerotic plaque burden, possibly due to the relatively short intervention period.

In a similar study conducted in an animal model, Rakipovski et al. demonstrated that long‐term treatment (12–17 weeks) with liraglutide and semaglutide reduced plaque progression independently of weight loss or lipid lowering [86]. Furthermore, in a model of acute aortic wall inflammation induced by lipopolysaccharide (LPS), semaglutide reduced TNF‐*α*, IL‐6, interleukin‐1 receptor antagonist (IL‐1RN), and MCP‐1 levels.

Several studies have further explained the anti‐inflammatory effects of GLP‐1 RAs through modulation of signaling pathways such as NF‐*κ*B, AMPK‐Sirt1, and RMRP/miR‐128‐1‐5P/Gadd45g [87, 88]. In contrast to these experimental findings, a clinical study involving patients with T2D and low to intermediate CV risk found that liraglutide did not reduce vascular inflammation, as assessed by 18F‐fluorodeoxyglucose PET imaging after 26 weeks of therapy [89]. This result may suggest that the anti‐inflammatory effects of GLP‐1 RAs are more pronounced in patients with advanced or high cardiovascular risk.

### 3.2. GLP‐1–Mediated Regulation of Endothelial Signaling Pathways

Endothelial activation followed by dysfunction is an integral component of atherogenesis, allowing inflammatory cells and plaque‐derived debris to penetrate into the intimal layers of the vessel wall. Under the influence of agents such as tobacco smoke constituents, proinflammatory cytokines, ROS, AGEs, and oxidized low‐density lipoprotein (ox‐LDL), the endothelium becomes more permeable to leukocytes and acquires prothrombotic properties—initiating the formation of atherosclerotic plaques [24, 90, 91]. Activated endothelial cells increase the surface expression of selectins and adhesion molecules, promoting leukocyte and platelet rolling, activation, and further propagation of inflammation.

Oxidized LDL, via its receptor lectin‐like oxidized LDL receptor‐1 (LOX‐1), has become a potential therapeutic target. A clinical trial with an anti–LOX‐1 monoclonal antibody is currently underway [92]. In vitro studies using human aortic endothelial cells (HAECs) exposed to ox‐LDL demonstrated that liraglutide may exert a protective effect on the transcription factor Krüppel‐like factor 2 (KLF2) via a mitogen‐activated protein kinase/extracellular signal–regulated kinase 5 (MAPK‐ERK5)–dependent pathway [93]. KLF2 is a key regulator of vascular endothelial biology, modulating endothelial nitric oxide synthase (eNOS), occludin expression, and NF‐*κ*B pathway regulation [94]. Studies on HUVECs, a standard experimental model of the endothelium, have also shown that liraglutide reduces LOX‐1 expression in cells exposed to ox‐LDL [95]. These findings suggest that GLP‐1 RAs may inhibit LOX‐1–mediated endothelial activation. Additional mechanisms have been proposed, including inhibition of PTEN‐induced kinase 1 (PINK1)/Parkin‐dependent mitophagy [96]. HUVECs cultured under hyperglycemic conditions exhibited mitochondrial membrane potential preservation and increased eNOS phosphorylation when treated with liraglutide. A synergistic effect of liraglutide and metformin was also studied in HUVECs, where an antiproliferative effect on endothelial cells was observed, suggesting possible beneficial roles of GLP‐1 RAs in oncology [97].

Endothelial adhesion molecule expression, a contributor to atherogenesis, has also become a focus of interest in the context of GLP‐1 therapy. In addition to molecular and immunoassay‐based evaluations of adhesion molecules, contrast‐enhanced ultrasound molecular imaging (CEUMI) has been used. In one such in vivo study in ApoE−/− mice, contrast microbubbles targeted to vascular cell adhesion molecule‐1 (VCAM‐1) showed a threefold signal increase in the control group, whereas liraglutide treatment prevented this response, maintaining stable VCAM‐1 levels throughout the study [98]. This study also reported reductions in TNF‐*α*, IL‐1*β*, and MCP‐1 concentrations following treatment, independently of glucose levels; however, no analyses were performed to assess whether these effects correlated with weight loss.

Similar results were found in an earlier 2011 study using immunohistochemistry in an atherosclerotic mouse model, where liraglutide reduced the expression of intercellular adhesion molecule‐1 (ICAM‐1), whereas no significant changes in blood pressure or body weight were observed in the treatment groups [99]. These effects were hypothesized to be mediated by inhibition of TNF‐*α* through GLP‐1R activation. To further elucidate mechanisms, Krasner et al. demonstrated in HAEC cultures that liraglutide downregulated E‐selectin and VCAM‐1 expression via increased intracellular Ca^2+^ levels, activation of Ca^2+^/calmodulin‐dependent protein kinase kinase‐*β* (CaMKK*β*), and subsequent AMPK activation—leading to suppression of the inflammatory response [100].

Another clinical study assessed the effects of liraglutide in obese women with and without polycystic ovary syndrome (PCOS) [101]. After 6 months of treatment, a similar reduction in soluble P‐selectin, ICAM‐1, and VCAM‐1 levels was observed in both groups, despite only moderate weight loss of 3%–4%. However, the absence of a placebo‐controlled group with similar weight loss made it difficult to determine whether these effects were drug‐specific or weight loss–related [101].

Conflicting data came from a study involving patients with T2D at high CV risk, where a 26‐week treatment with semaglutide or dulaglutide resulted in increased serum ICAM‐1 levels but decreased concentrations of L‐selectin and MCP‐1 [80]. No correlation with changes in body weight was observed; however, this study did not adequately account for the influence of hyperglycemia or concurrent medications, limiting the interpretability of the findings.

### 3.3. Influence on Endothelial Function Regulation via NO

The enzymes eNOS and iNOS (inducible nitric oxide synthase) play a crucial role in regulating endothelial function and act as key mediators of vascular tone and homeostasis through the generation of NO [102]. Numerous animal studies have provided evidence of the impact of GLP‐1 RAs on this aspect of vascular endothelial function.

In the previously mentioned 2011 study, immunohistochemical analysis of aortic rings from ApoE−/− mice demonstrated a significant increase in eNOS expression in the endothelium following liraglutide treatment—a response that was abolished when coadministered with the GLP‐1R antagonist exendin‐9 [99]. Han et al. [103] showed in a model of obese mice that liraglutide improved endothelial function via activation of the AMPK/eNOS signaling pathway, a mechanism further confirmed in experimental studies using HAECs [100].

Other in vitro models have suggested that GLP‐1 RAs act via the Janus Kinase 2/signal transducer and activator of transcription 3 (JAK2/STAT3) signaling pathway, resulting in increased eNOS expression and angiogenic factor production—mechanisms that may contribute to their anti‐ischemic effects [104]. Liraglutide has also been shown to enhance eNOS phosphorylation and increase NO production, an effect at least partially dependent on activation of the mechanistic target of rapamycin (mTOR) signaling pathway [105]. This pathway is one of the core regulators of cellular metabolism. Additionally, studies have shown a protective effect of GLP‐1 RAs on pancreatic beta cells, cardiomyocytes, and neurons via the mTOR pathway, suggesting that mTOR signaling may be a central mechanism of action for this drug class [106–108].

In contrast to eNOS, GLP‐1 RAs appear to exert an inhibitory effect on iNOS. Reduced iNOS expression within atherosclerotic plaques is associated with diminished vascular inflammation and increased plaque stability [109, 110]. In murine models of atherosclerosis, intervention with lixisenatide led to decreased iNOS expression and increased Arginase I content within the plaques, indicating attenuated proinflammatory signaling and reduced oxidative stress [83]. As mentioned above, these results should be interpreted cautiously, as lixisenatide did not demonstrate cardiovascular risk reduction in CVOTs [84]. The positive effect of liraglutide on iNOS‐dependent nitro‐oxidative stress was also demonstrated in another animal model, suggesting that this effect is partly mediated through the AMPK pathway [111].

### 3.4. Influence on MMPs

MMPs are a family of zinc‐dependent endopeptidases responsible for tissue remodeling and the degradation of extracellular matrix proteins. MMPs can modulate various cellular and signaling pathways involved in atherosclerosis, particularly those associated with plaque progression and rupture [112]. Their secretion is regulated by proinflammatory cytokines, as well as by specific endogenous tissue inhibitors (tissue inhibitors of metalloproteinases—TIMPs) [113].

The role of MMP‐9 in the development of atherosclerosis and endothelial dysfunction has been confirmed in multiple studies [114–116]. Several ex vivo investigations using animal vessels have demonstrated that GLP‐1 RAs reduce MMP‐9 levels [117–119]. In human coronary artery smooth muscle cells (hCASMCs) exposed to inflammatory stimuli, exenatide was shown to reduce the expression of MMP‐1, MMP‐2, and MMP‐9, likely through inhibition of the Akt signaling pathway [120]. Liraglutide has also been shown to upregulate the expression of TIMP‐2, which may represent an additional mechanism of action for this drug class [85].

In a murine model of atherosclerosis, GLP‐1 RAs reduced MMP‐9 expression, limited macrophage infiltration, and increased collagen content and fibrous cap thickness within plaques—changes associated with enhanced plaque stability [121]. However, the molecular mechanisms through which GLP‐1 RAs modulate MMP expression remain poorly understood, and there is a lack of clinical data directly linking GLP‐1 RAs with MMP modulation. Given the close association between MMPs and inflammatory signaling pathways, it is likely that the observed effects are mediated indirectly via the anti‐inflammatory properties of GLP‐1 RAs.

### 3.5. Influence on VSMCs

A characteristic feature of advanced atherosclerotic lesions is an enhanced prothrombotic state and remodeling of deeper vascular wall layers, including VSMCs [122, 123]. Although these processes are primarily associated with later stages of atherogenesis, increased VSMCs proliferation and migration—secondary to MMP activity—have also been demonstrated during earlier stages [124, 125].

A key aspect of pathological VSMCs transformation is the phenotypic switch from a contractile to a so‐called synthetic phenotype [122]. Studies using cultured VSMCs have shown that liraglutide inhibits VSMCs proliferation, migration, and phenotypic switching by suppressing the expression of proteins involved in lipid metabolism, such as PCSK9 and the LDL receptor [126]. Another study, using VSMC cultures under hyperglycemic conditions, reported a similar effect mediated through inhibition of the ERK1/2 and PI3K/Akt signaling pathways [127].

Liraglutide has also been shown to suppress AGE‐induced phenotypic switching of VSMCs. GLP‐1 RAs increased the expression of contractile VSMC markers while reducing collagen production, likely through downregulation of myocardin, inhibition of NF‐*κ*B signaling, and activation of the PKA signaling pathway [128]. In another VSMC culture model, exendin‐4 was found to act via AMPK/Sirtuin 1/Forkhead Box O3a (AMPK/SIRT1/FOXO3a) signaling, leading to increased expression of contractile VSMC markers such as calponin and SM22*α* [129].

In contrast to these findings, Bjørnholm et al. reported a reduction in the expression of two contractile phenotype markers—Cnn1 and Cald1—in atherosclerotic vascular walls of animals treated with liraglutide for 4 weeks [85]. Since the contractile phenotype of VSMCs is considered physiological, these results may suggest a potentially adverse effect of GLP‐1 RAs. However, the role of VSMCs hypertrophy and phenotypic switching remains debated, as this transformation may actually promote fibrous cap formation and plaque stabilization, thereby reducing cardiovascular risk [122]. This hypothesis is supported by a study conducted in Watanabe heritable hyperlipidemic rabbits, where 12 weeks of liraglutide therapy led to increased fibrosis of VSMCs, as demonstrated by histopathological analysis of atherosclerotic arterial walls—suggesting enhanced plaque stability [130].

### 3.6. Effects on Hemostasis and Coagulation

The association between plasminogen activator inhibitor‐1 (PAI‐1) and atherothrombosis is well established. Elevated levels of PAI‐1 are a recognized risk factor for thrombosis and vascular complications in diabetes [131]. Increased PAI‐1 concentrations have also been observed in patients with subclinical atherosclerotic changes [132]. This molecule is a key inhibitor of fibrinolysis, leading to pathological fibrin deposition and thrombus formation [133].

Initial studies on the effects of GLP‐1 RAs on PAI‐1 expression, conducted in HUVECs and dating back to 2009, demonstrated inhibition of PAI‐1 induction—suggesting a mechanism involving TNF‐*α* modulation via the Akt signaling pathway and the nuclear Receptor Nur77 [134, 135]. However, subsequent studies in human populations suggest that the inhibition of PAI‐1 expression by liraglutide may be indirect, resulting from improvements in hyperglycemia or inflammation, and is likely not driven by weight loss or improved IR [136].

A meta‐analysis of GLP‐1–based therapies in patients with T2D showed a minor reduction in serum PAI‐1 levels, with a mean difference of approximately −12.9% (*p* = 0.05), indicating a modest but consistent lowering effect in this population [137]. Further studies are warranted, particularly those conducted in larger patient cohorts under conditions that eliminate the confounding effects (e.g. hyperglycemia), to better elucidate the mechanisms underlying PAI‐1 downregulation by GLP‐1 RAs.

## 4. Summary

This review presents the current state of knowledge regarding the potential antiatherosclerotic mechanisms of GLP‐1 RAs. In light of the recently proposed concept of the CKM syndrome, it is clear that CV risk is driven by a complex interplay of multiple factors [51]. According to this framework, GLP‐1 RAs reduce CV risk indirectly through beneficial effects on adipose tissue, glycemic control, lipid metabolism, blood pressure, and kidney function.

CVOTs such as LEADER, SUSTAIN‐6, REWIND, and SELECT have consistently demonstrated that GLP‐1 RAs reduce MACE in patients with T2D and, importantly, also in individuals without diabetes. This finding suggests that the cardioprotective effects of GLP‐1 RAs extend beyond classical glycemic control and involve multiple mechanisms beneficial to the cardiovascular system, including anti‐inflammatory activity, endothelial function improvement, oxidative stress modulation, and vascular remodeling. Considering the relationship between these factors and obesity, further studies are required to determine the magnitude of the effect of GLP‐1 RAs on these parameters mediated by body‐weight reduction.

GLP‐1 RAs have been shown to inhibit foam cell formation, reduce the expression of proinflammatory cytokines, and modulate macrophage polarization from a proinflammatory M1 phenotype to an anti‐inflammatory M2 phenotype. These effects have been confirmed in numerous animal models; however, data from human studies remain partially inconsistent. Some clinical trials have failed to show significant reductions in vascular inflammation following GLP‐1 RA therapy, potentially due to short treatment durations, low baseline CVD risk, or confounding pharmacotherapy.

Another key area of interest is the effect of GLP‐1 RAs on the vascular endothelium. In vitro and in vivo evidence suggests improvements in endothelial function via activation of the AMPK/eNOS pathway, downregulation of adhesion molecules and LOX‐1, and reduction of oxidative stress. As with the anti‐inflammatory effects, much of this evidence is based on experimental data, limiting its direct translation to clinical practice. Given the influence of multiple factors on inflammation and vascular endothelial activity (e.g., cigarette smoking and concomitant medications), further functional research in human cohorts is necessary to account for these confounders and to establish a definitive cause‐and‐effect relationship.

GLP‐1 RAs also influence VSMC activity by modulating migration, proliferation, and phenotypic switching. These actions may contribute to plaque stabilization, although the clinical relevance of promoting the contractile over the synthetic VSMC phenotype remains debated. In addition, some studies suggest that GLP‐1 RAs may reduce MMPs′ activity, potentially limiting plaque remodeling and the risk of rupture.

With regard to the coagulation system, a noteworthy finding is the suppression of PAI‐1 expression—a major inhibitor of fibrinolysis and a recognized marker of thrombotic risk. Although the molecular mechanisms underlying this effect are not fully understood, available data indicate a favorable impact of GLP‐1 RAs on prothrombotic status, which plays a critical role in cardiovascular events.

**Figure 1:**
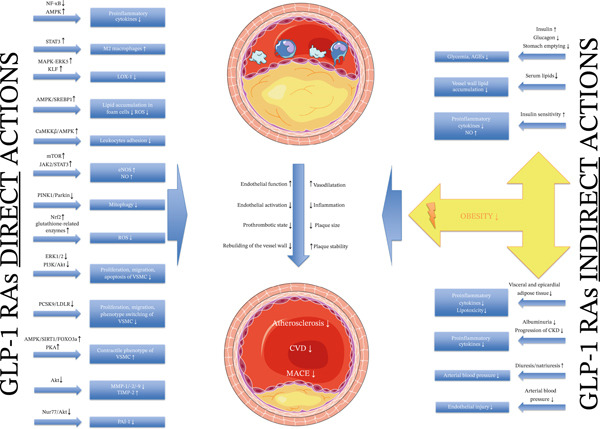
Suggested mechanisms of the antiatherogenic effects of glucagon‐like peptide‐1 receptor agonists. † AGE—advanced glycation end‐product; Akt—protein kinase B; AMPK—AMP‐activated protein kinase; CaMKK*β*—calcium/calmodulin‐dependent protein kinase kinase *β*; CKD—chronic kidney disease; CVD—cardiovascular disease; eNOS—endothelial nitric oxide synthase; ERK—extracellular signal‐regulated kinase; FOXO3a—Forkhead Box O3; GLP‐1 RA—glucagon‐like peptide‐1 receptor agonist; iNOS—inducible nitric oxide synthase; JAK—Janus kinase; KLF—Krüppel‐like factor; LDLR—low‐density lipoprotein receptor; LOX‐1—lectin‐like oxidized low‐density lipoprotein receptor‐1; MACE—major adverse cardiovascular event; MAPK—mitogen‐activated protein kinase; mTOR—mammalian target of rapamycin; NF‐*κ*B—nuclear factor kappa B; NO—nitric oxide; Nrf 2—nuclear transcription factor erythroid 2p45‐related factor; Nur77—nuclear receptor subfamily 4 group A member 1; PAI‐1—plasminogen activator inhibitor‐1; PCSK9—proprotein convertase subtilisin/kexin type 9; PINK1—PTEN‐induced kinase 1; PKA—protein kinase A; ROS—reactive oxygen species; SIRT1—Sirtuin 1, SREBP1—sterol regulatory element‐binding protein 1; STAT—signal transducer and activator of transcription; TIMP‐2—tissue inhibitor of metalloproteinases‐2; VSMC—vascular smooth muscle cell. ‡ Images are adapted from Servier Medical Art (https://smart.servier.com/), licensed under CC BY 4.0 (https://creativecommons.org/licenses/by/4.0/).

Taken together, these findings support the hypothesis of a complex, multifactorial antiatherosclerotic mechanism of GLP‐1 RAs (as summarized in Figure 1). However, a major limitation is that much of the current evidence stems from experimental studies or small clinical trials lacking proper control groups (see Table 1). Future randomized functional studies involving broader and more diverse patient populations—including both diabetic and nondiabetic individuals—are needed to better elucidate the direct effects of GLP‐1 RAs on atherogenesis.

**Table 1:** Summary of cited studies on the direct antiatherogenic mechanisms of glucagon‐like peptide‐1 receptor agonists.

Authors	Reference	Study type	Agent	Duration of treatment	Model/population	Key findings and potential mechanisms
Arakawa et al. (2010)	[74]	Preclinical	Exendin‐4	4 weeks	ApoE−/− mice	Suppression of monocyte adhesion to the endothelium and attenuation of atherosclerotic lesions.
Bray et al. (2021)	[76]	Meta‐analysis	Liraglutide, semaglutide, exenatide	Various	Patients with T2D	Reduction in oxidative stress biomarker levels (MDA), potentially through mechanisms involving improved insulin resistance and blood pressure regulation independent of glycemic control.
Li et al. (2017)	[77]	Preclinical	GLP‐1	—	HUVECs	Reduction of high‐glucose–induced oxidative stress by inhibiting NOX4, p47phox, and Rac‐1 expression and translocation of p47phox.
Fernández‐Millán et al. (2016)	[78]	Preclinical	GLP‐1	—	Rat INS‐1E cells (pancreas)	Increase in synthesis of glutathione‐related enzymes and activating the PKA‐dependent ERK/Nrf2 signaling pathway–antioxidative effects.
Wang et al. (2015)	[79]	Preclinical	Liraglutide	—	Raw 264.7 cells (macrophages)	Attenuation of foam cell formation and oxidative stress via AMPK/SREBP1 signaling.
Hahuła et al. (2024)	[80]	Clinical	Dulaglutide/semaglutide	26 weeks	Patients with T2D	Reduction in MCP‐1 and L‐selectin levels, increase in serum ICAM‐1 level.
Hou et al. (2024)	[82]	Preclinical	Liraglutide/dulaglutide	—	Murine macrophages/HUVECs	Promotion of M2 macrophage polarization and reduction of IL‐1*β* and TNF‐*α* expression.
Vinué et al. (2017)	[83]	Preclinical	Liraglutide/lixisenatide	12 weeks	Apoe −/− and Irs2 +/− mice	Decrease in iNOS expression and increase in Arginase I content within the plaques; enhanced activation of STAT3—a key factor in promoting M2 macrophage differentiation.
Bjørnholm et al. (2021)	[85]	Preclinical	Liraglutide	4 weeks	LDL‐R −/− mice	Reduction in CCR5, fractalkine, and MCP‐1 levels.
Rakipovski et al. (2018)	[86]	Preclinical	Liraglutide/semaglutide	12–17 weeks	LDL‐R−/− and ApoE−/− mice	Reduction of plaque progression, reduction in TNF‐*α*, IL‐6, IL‐1RN, and MCP‐1 levels.
Zhou et al. (2019)	[87]	Preclinical	Liraglutide	2 weeks	HFD‐induced diabetic mice	Reduction in circulating IL‐1*β* and IL‐6 levels, as well as hepatic IL‐1*β* and IL‐6 content, reduction in the expression of iNOS‐1 and COX‐2 in insulin‐sensitive tissues following liraglutide treatment, and activation of AMPK‐*α* and Sirt‐1.
An et al. (2020)	[88]	Preclinical	Liraglutide	—	Rat model of coronary atherosclerosis and HVSMCs	Regulation of the lncRNA RMRP/miR‐128‐1‐5P/Gadd45g axis resulting in inhibition of IL‐6 and IL‐8 expression.
Yue et al. (2019)	[93]	Preclinical	Liraglutide	—	HAECs	Protective effect on KLF2 via a MAPK‐ERK5–dependent pathway, resulting in inhibition of LOX‐1 activation.
Ying et al. (2023)	[95]	Preclinical	Liraglutide	—	HUVECs	Reduction of LOX‐1 expression in cells exposed to ox‐LDL.
Zhang et al. (2022)	[96]	Preclinical	Liraglutide	—	HUVECs	Prevention of high glucose–induced endothelial dysfunction via inhibition of PINK1/Parkin‐dependent mitophagy.
Shadboorestan et al. (2022)	[97]	Preclinical	Liraglutide + metformin	—	HUVECs	Antiproliferative effect on endothelial cells .
Punjabi et al. (2023)	[98]	Preclinical	Liraglutide	8–12 weeks	ApoE−/− mice	Prevention of the adhesion molecule response, maintenance of stable VCAM‐1 levels throughout the study, and reductions in TNF‐*α*, IL‐1*β*, and MCP‐1 levels
Gaspari et al. (2011)	[99]	Preclinical	Liraglutide	12 weeks	ApoE−/− mice	Reduction in ICAM‐1 and TNF‐*α* expression, improvement in endothelium‐dependent relaxation of aortic rings, and a significant increase in eNOS expression.
Krasner et al. (2014)	[100]	Preclinical	Liraglutide	—	HAECs	Downregulation of E‐selectin and VCAM‐1 via activation of the CaMKK*β*/AMPK pathway.
Kahal et al. (2015)	[101]	Clinical	Liraglutide	24 weeks	Obese young women with PCOS	Reduction in soluble P‐selectin, ICAM‐1, and VCAM‐1 levels
Han et al. (2019)	[103]	Preclinical	Liraglutide	8 weeks	HFD‐induced obese mice	Improvement of endothelial function (EDV) via activation of the AMPK/eNOS signaling pathway.
Di et al. (2020)	[104]	Preclinical	Liraglutide	—	HUVECs	JAK2/STAT3 signaling pathway‐mediated increase in eNOS expression and angiogenic factor production.
Wu et al. (2021)	[105]	Preclinical	Liraglutide	—	HUVECs	mTOR activation‐dependent enhancement of eNOS phosphorylation and NO production.
Miao et al. (2012)	[106]	Preclinical	Liraglutide	—	INS‐1 beta‐cell (pancreas)	AMPK/mTOR/P70S6K signaling pathway‐mediated regulation of pancreatic beta‐cell proliferation and apoptosis.
Yu et al. (2018)	[107]	Preclinical	Exendin‐4/liraglutide	—	Cardiomyocytes isolated from adult mice and H9c2 myoblast cells	Attenuation of glucose toxicity–induced cardiac injury through mTOR/ULK1‐dependent autophagy.
Kimura et al. (2013)	[108]	Preclinical	GLP‐1	—	PC12 cells (neuroblastic cells)	GLP‐1–mediated neuroprotection against MG‐induced apoptosis via EGFR transactivation and PI3K/Akt/mTOR/GCLc/redox signaling.
Steven et al. (2017)	[111]	Preclinical	Liraglutide	72 h	DPP‐4−/−, GLP‐1r−/− mice	Reduction in microvascular thrombosis, nitro‐oxidative stress, and platelet activation.
Ding et al. (2022)	[117]	Preclinical	Liraglutide	4 weeks	HFD ‐induced diabetic rabbits	Reduction in expression of PCNA, MMP‐9.
Kim et al. (2021)	[118]	Preclinical	Liraglutide/FGF21	6 weeks	HFD‐induced diabetic rabbits	Caspase‐3 increase with concomitant MMP‐9, ICAM‐1, p‐Akt, and p‐ERK1/2 downregulation.
Zhao et al. (2024)	[119]	Preclinical	Liraglutide	4 weeks	Mice with inducted aortic aneurysm	Reduction in MDA expression and MMP‐2/‐9 activity.
Gallego‐Colon et al. (2018)	[120]	Preclinical	Exendin‐4/GLP‐1	—	hCASMC	AKT‐Thr308 phosphorylation inhibition‐mediated modulation of MMP‐1, ‐2, and ‐9 expression.
Burgmaier et al. (2013)	[121]	Preclinical	GLP‐1 and split products	12 weeks	ApoE−/− mice	Reduction in plaque macrophage infiltration, MMP‐9 expression, and plaque collagen content.
Ji et al. (2021)	[126]	Preclinical	Liraglutide	—	hCASMCs	Inhibition of VSMC proliferation, migration, and phenotypic switching through suppression of lipid metabolism proteins, including PCSK9 and LDL‐R.
Shi et al. (2015)	[127]	Preclinical	Liraglutide	—	Rat VSMCs	Attenuation of high glucose–induced VSMC abnormal migration, proliferation, and apoptosis. Inhibition of VSMC phenotypic switching via GLP‐1R‐dependent PI3K and ERK1/2 activation.
Di et al. (2019)	[128]	Preclinical	Liraglutide	—	Rat CASMCs	Inhibition of AGE‐induced SMC phenotypic transition, increased contractile marker expression, and decreased collagen production via myocardin downregulation, NF‐*κ*B inhibition, and PKA activation.
Liu et al. (2018)	[129]	Preclinical	Exendin‐4	—	Rat VSMCs	Regulation of VSMC phenotype switching and promotion of redifferentiation via AMPK/SIRT1/FOXO3a signaling pathways.
Sudo et al. (2017)	[130]	Preclinical	GLP‐1	12 weeks	Watanabe heritable hyperlipidemic (WHHL) rabbits	Increased VSMC fibrosis suggesting enhanced plaque stability.
Liu et al. (2009)	[134]	Preclinical	Liraglutide	—	HUVECs	NUR77 modulation‐mediated inhibition of TNF‐ or glucose‐induced PAI‐1 and vascular adhesion molecule expression.
Liu et al. (2008)	[135]	Preclinical	GLP‐1	—	HUVECs	Inhibition of TNF‐*α*–mediated PAI‐1 induction in vascular endothelial cells via potential Akt signaling and Nur77 modulation.
Mashayekhi et al. (2023)	[136]	Clinical	Liraglutide	14 weeks	Individuals with obesity and prediabetes	Reduction in serum PAI‐1 and MCP‐1 levels.
Song et al. (2015)	[137]	Meta‐analysis	Exenatide, liraglutide, albiglutide, dulaglutide, lixisenatide	Various	Patients with T2D	Reduction in serum PAI‐1 levels.

Abbreviations: AGEs, advanced glycation end‐products; AMPK, AMP‐activated protein kinase; ApoE−/−, apolipoprotein E‐deficient; CaMKK*β*, calcium/calmodulin‐dependent protein kinase kinase beta; CASMCs, coronary artery smooth muscle cells; CCR5, C‐C chemokine receptor Type 5; COX‐2, cyclooxygenase‐2; DPP‐4−/−, dipeptidyl peptidase‐4‐deficient; EDV, endothelium‐dependent vasodilation; EGFR, epidermal growth factor receptor; eNOS, endothelial nitric oxide synthase; ERK, extracellular signal‐regulated kinase; FOXO3a, Forkhead Box transcription Factor O3a; GADD45g, growth arrest and DNA damage‐inducible protein gamma; GCLc, glutamate‐cysteine ligase catalytic subunit; GLP‐1, glucagon‐like peptide‐1; GLP‐1R/GLP‐1r−/−, glucagon‐like peptide‐1 receptor/GLP‐1 receptor‐deficient; HAECs, human aortic endothelial cells; hCASMC, human coronary artery smooth muscle cell; HFD, high‐fat diet; HUVECs, human umbilical vein endothelial cells; HVSMCs, human vascular smooth muscle cells; ICAM‐1, intercellular adhesion molecule‐1; IL‐1*β*/IL‐6/IL‐8, interleukin‐1 beta/6/8; IL‐1RN, interleukin‐1 receptor antagonist; iNOS, inducible nitric oxide synthase; IRS2+/−, insulin receptor substrate 2‐heterozygous; JAK2, Janus Kinase 2; KLF2, Krüppel‐like Factor 2; LDL‐R/LDL‐R−/−, low‐density lipoprotein receptor/LDL receptor‐deficient; lncRNA, long noncoding RNA; LOX‐1, lectin‐like oxidized LDL receptor‐1; MAPK, mitogen‐activated protein kinase; MCP‐1, monocyte chemoattractant protein‐1; MDA, malondialdehyde; MG, methylglyoxal; MMP‐1/‐2/‐9, matrix metalloproteinase‐1/‐2/‐9; mTOR, mechanistic target of rapamycin; NCF1, p47phox neutrophil cytosol Factor 1; NF‐*κ*B, nuclear factor kappa‐light‐chain‐enhancer of activated B cells; NO, nitric oxide; NOX4, NADPH Oxidase 4; Nrf2, nuclear factor erythroid 2‐related factor 2; Nur77, orphan nuclear Receptor NR4A1; ox‐LDL, oxidized low‐density lipoprotein; P70S6K, ribosomal protein S6 kinase beta‐1; PAI‐1, plasmonogen activator inhibitor‐1; PCNA, proliferating cell nuclear antigen; PCOS, polycystic ovary syndrome; PCSK9, proprotein convertase subtilisin/kexin Type 9; PI3K, phosphoinositide 3‐kinase; PINK1, PTEN‐induced Kinase 1; PKA, protein kinase A; PKB, Akt‐protein kinase B; Rac‐1, Ras‐related C3 botulinum toxin Substrate 1; RMRP, RNA component of mitochondrial RNA‐processing endoribonuclease; SIRT1, Sirtuin 1; SMC/VSMC, (vascular) smooth muscle cell; SREBP1, sterol regulatory element‐binding Protein 1; STAT3, signal transducer and activator of transcription 3; T2D, Type 2 diabetes; TNF‐*α*, tumor necrosis factor alpha; ULK1, Unc‐51 like autophagy activating Kinase 1; VCAM‐1, vascular cell adhesion molecule‐1.

## Funding

No funding was received for this manuscript.

## Conflicts of Interest

The authors declare no conflicts of interest.

## Data Availability

Data sharing is not applicable to this article as no datasets were generated or analyzed during the current study.
